# Polycomb Repressive Complex 2 Targets Murine Cytomegalovirus Chromatin for Modification and Associates with Viral Replication Centers

**DOI:** 10.1371/journal.pone.0029410

**Published:** 2012-01-18

**Authors:** Christopher G. Abraham, Caroline A. Kulesza

**Affiliations:** Department of Microbiology, University of Colorado School of Medicine, Aurora, Colorado, United States of America; Washington University School of Medicine, United States of America

## Abstract

Regulation of viral transcription by chromatin structure has emerged as a fundamental determinant in the establishment of lytic and latent herpesvirus infections. The Polycomb group (PcG) of epigenetic repressors promotes heterochromatin formation by trimethylating histone H3 on lysine-27 (H3K27me3) and regulates development, stem cell renewal and differentiation and the cell cycle. These cellular processes are tightly coupled to the molecular switch between lytic and latent herpesvirus infections. Using chromatin immunoprecipitation analysis, we observed enrichment of H3K27me3 at the major immediate-early (MIE) locus of murine cytomegalovirus (MCMV) very early following infection of permissive fibroblasts. As lytic replication progressed, we observed a loss of H3K27me3 enrichment concomitant with the appearance of H3K4me3. However, late during infection, as viral replication centers are established, we observed a significant increase in PcG protein association with chromatin. Additionally, in co-immunofluorescence assays using confocal microscopy, we detected strong enrichments for PcG protein within the viral replication compartment, suggesting an association between viral DNA synthesis machinery and PcG proteins. Together, our results suggest a novel, dynamic interaction between PcG epigenetic repressors and MCMV genomes.

## Introduction

Human cytomegalovirus (HCMV) is a β-herpesvirus and a ubiquitous human pathogen [1]. HCMV infections are frequently associated with opportunistic disease in immunocompromised hosts, such as AIDS patients, cancer patients and bone marrow and solid organ transplant recipients. Transplacental transmission of HCMV from mother to fetus is also a major concern, as it can result in serious sequelae, ranging from sensorineural hearing loss and developmental deficits to death. Like all herpesviruses, HCMV persists for the lifetime of the host, maintaining a latent infection in hematopoietic progenitor cells of the myeloid lineage [2]. Reactivation of latent virus to acute replication promotes transmission, causes opportunistic disease in immunocompromised hosts, and likely promotes reseeding of the latent reservoir.

The lytic and latent phases of HCMV replication depend upon a highly regulated program of viral gene expression that relies on the coordinated interaction of viral and cellular factors. During HCMV replication in permissive cells, viral genes are expressed in a temporal cascade. Immediate early (IE) genes are expressed from the genome first, independent of any other gene expression. Expression of viral Early (E) and Late (L) genes subsequently follows and depends upon robust IE gene expression. Protein products of the HCMV Major Immediate Early Promoter (MIEP), namely IE1-72 and IE2-86, play a central role in driving the acute replication cycle, as well as promoting reactivation from latency [3]. As there is no animal model for HCMV infection, murine cytomegalovirus (MCMV) is widely used as a model system to study the regulation of β-herpesvirus gene expression both *in vitro* and *in vivo*. Studies of cytomegalovirus infections suggest that control of MIEP activity and, consequently, expression levels of IE proteins, is a critical determinant of whether the lytic or latent transcriptional program is executed [4], [5]. Both HCMV and MCMV MIE enhancer/promoter regions are complex, transcriptional regulatory modules, rich in *cis*-acting regulatory elements. These sequences are recognized by both positive and negative cellular transcriptional regulators (i.e. YY1, CREB/ATF and NF-κB), whose functions are regulated by both virus and host factors. Tertiary chromatin structure also contributes to MIE enhancer/promoter regulation, where heterochromatin specifies transcriptional quiescence during latency, and euchromatin permits transcriptional activation of the region during acute infections and reactivation. Identifying the factors that control this molecular switch between chromatin states is crucial to understanding the mechanisms governing acute and latent infections.

DNA-templated processes in eukaryotic cells, such as transcription, replication and repair, are universally influenced by chromatin structure. The basic building block of all chromatin is the nucleosome and the amino-terminal tails of all four core histones are subject to a myriad of post-translational modifications (PTMs) [6]. This “histone code” allows for the generation of distinct epigenetic micro-environments by altering nucleosome properties and chromatin compaction states, and by recruiting trans-acting factors to establish or maintain various chromatin domains [7]. There are two basic chromatin states: less-condensed, transcriptionally active euchromatin (EC) and highly-condensed, transcriptionally silent heterochromatin (HC) [8]. HC is further subdivided into constitutive or facultative heterochromatin (cHC or fHC). cHC is found at highly condensed genomic regions such as centromeres, telomeres and highly repetitive transposable elements, and lacks the potential to revert to transcriptionally active EC [9]. On the other hand, fHC, though transcriptionally silent, retains the potential to convert to transcriptionally active EC. Reversibly silenced fHC domains span genes often required for cell cycle regulation or developmental processes, such as the INK4a locus and the HOX genes [10], [11]. While the establishment and maintenance of fHC depends on a number of cellular processes, post-translational modifications of histones are of primary importance in the recognition and function of fHC. fHC is characterized by enrichment of histone H3 trimethylated on lysine 27 (H3K27me3) and to a lesser extent, with histone H3 di- and trimethylated on lysine 9 (H3K9me2/3). H3K27 trimethylation is catalyzed by the multi-protein effector complex, Polycomb Repressive Complex 2 (PRC2). EZH2 is the catalytic subunit of PRC2 and must bind to two other PRC2 subunits, SUZ12 and EED, for full activity. Additionally, while not required for PRC2 histone methyltransferase activity, the histone binding proteins RbAp46/48, and PHF1 are also PRC2 accessory subunits. In the establishment and maintenance of fHC, PRC2 cooperates with a second, functionally related complex known as PRC1 that modifies histone H2A on lysine 119 (H2AK119ub). This dual marking of nucleosomes is proposed to target chromatin for compaction and the exclusion of RNA polymerase, both critical to the maintenance of reversibly silenced chromatin [12]. The activities of the cellular Trithorax group proteins functionally oppose transcriptional repression by PRC1 and PRC2 and facilitate transcriptional activation [13]. Specifically, a Trithorax group protein known as KDM6A/B demethylates H3K27me3 while MLL1-3 catalyzes trimethylation of histone H3 on lysine-4 (H3K4me3), promoting transcriptional activation and elongation. As a result, cellular gene expression is dynamically regulated by the interplay of Polycomb group (PcG) and Trithorax group (TrxG) activities.

Like other herpesviruses, incoming cytomegalovirus genomes rapidly associate with cellular histone proteins upon entering the nucleus and numerous studies have confirmed the role chromatin structure plays in both the lytic and latent phases of cytomegalovirus infection [14], [15], [16], [17], [18], [19], [20]. Nucleosomes associate with the HCMV genome within 30 minutes after infection and are subject to various PTMs, generating an irregular chromatin structure on the genome [21]. Studies of both HCMV and MCMV have documented how changes in histone PTMs at IE, E and L promoters correlate with changes in gene expression during acute replication and latency [22], [23], [24], [25], [26], [27]. Some studies of HCMV and MCMV replication suggest that the chromatin structure established on viral genomes upon entering the nucleus initially serves to repress viral transcription through the formation of fHC marked by H3K9me2 [23], [24], [25]. Additionally, multiple studies support a role for PRCs in regulating α- and γ-herpesvirus gene expression and replication, however, for the β-herpesvirus sub-family, such an interaction has yet to be characterized. PcG proteins are known to play central roles in development, stem cell renewal and differentiation and the cell cycle, cellular processes that are also intimately linked to the molecular switch between latent and lytic cytomegalovirus infections [10], [11], [28], [29].

We hypothesized that fHC plays a significant role in the regulation of MCMV gene expression and analyzed the viral genome for evidence of histone PTMs characteristic of fHC. We measured a significant enrichment of the fHC mark H3K27me3 at the MIE locus of MCMV very early after infection. As the IE phase proceeds, enrichment of this mark decreases, with concomitant appearance of H3K4me3, a modification strongly associated with transcriptionally active chromatin [30], [31]. Since trimethylation of H3K27 is catalyzed by PRC2, and the presence of H3K27me3 decreases in the IE-to-E transition, we hypothesized that virus infection may actively antagonize PRC2 function to promote viral gene expression. In response to MCMV infection, we detected alterations in the nuclear staining of PRC2 proteins, dependent on the expression of IE proteins and genome replication. Additionally, we observed that infection resulted in an increase in chromatin-associated PRC2 and enrichment of PRC2 proteins in viral replication centers. Taken together, our results suggest that PRCs participate in the pre-IE repression of MCMV lytic expression, and that a viral determinant targets PRC2 to facilitate robust viral transcription and replication.

## Results

### H3K27me3 is enriched at the MCMV MIE locus at pre-immediate-early times of infection

**Figure 1:**
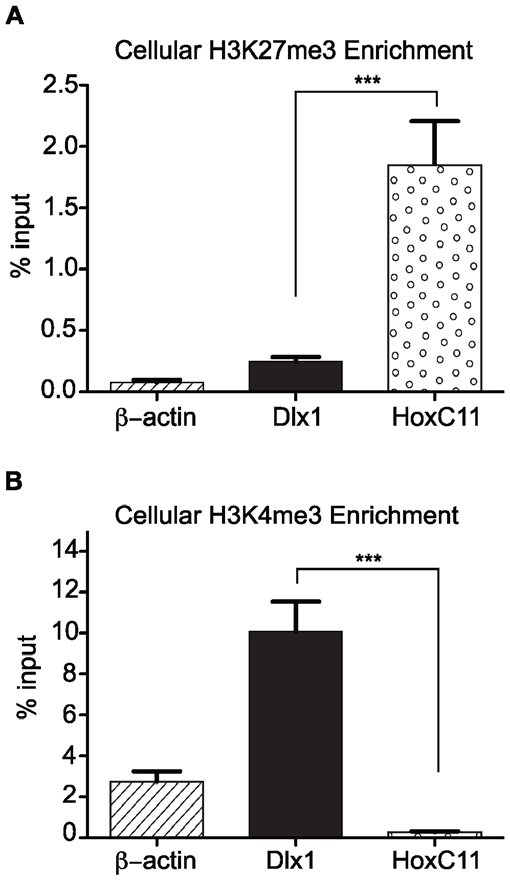
Validation of ChIPs for (A) H3K27me3 and (B) H3K4me3. Chromatin from uninfected mouse fibroblasts was immunoprecipitated using anti-H3K27me3 antibody. The specificity of the ChIPs was assessed by measuring the %Input by Q-PCR at HoxC11, Dlx1 and β-Actin. Each graph displays the mean value and S.E.M. for each queried locus, with data from four independent ChIPs. *** *P* value<0.001.

Trimethylation of histone H3 on lysine-27 (H3K27me3) is consistently associated with reversibly silenced domains of chromatin known as facultative heterochromatin (fHC) [8]. Recent studies have examined the association of repressive PTMs such as H3K9me2 as well as accessory proteins such as HP1β with cytomegalovirus promoters, yet none have examined the composition of viral chromatin with respect to PRC2-catalyzed H3K27me3-enrichment [23], [24], [25]. To determine if viral genomes become associated with H3K27me3, we performed ChIP analysis of chromatin prepared from mouse fibroblasts infected with MCMV. To demonstrate that our immunoprecipitations were specific and efficient, we first validated our ChIP samples by assessing the enrichment of H3K27me3 at two cellular promoters compared to the β-actin promoter, which is not expected to change in histone marks throughout infection. The cellular gene HoxC11 serves a positive control for H3K27me3-enrichment and is known to be silenced in a PRC-dependent manner in fibroblasts. The cellular gene Dlx1 serves as a negative control as it is transcriptionally active and is not targeted for PRC-mediated silencing [32]. We observed a 7.4-fold enrichment of H3K27me3 at the promoter of HoxC11 over the negative control, Dlx1 (*P* value<0.001; ANOVA) (Fig. 1A).

**Figure 2:**
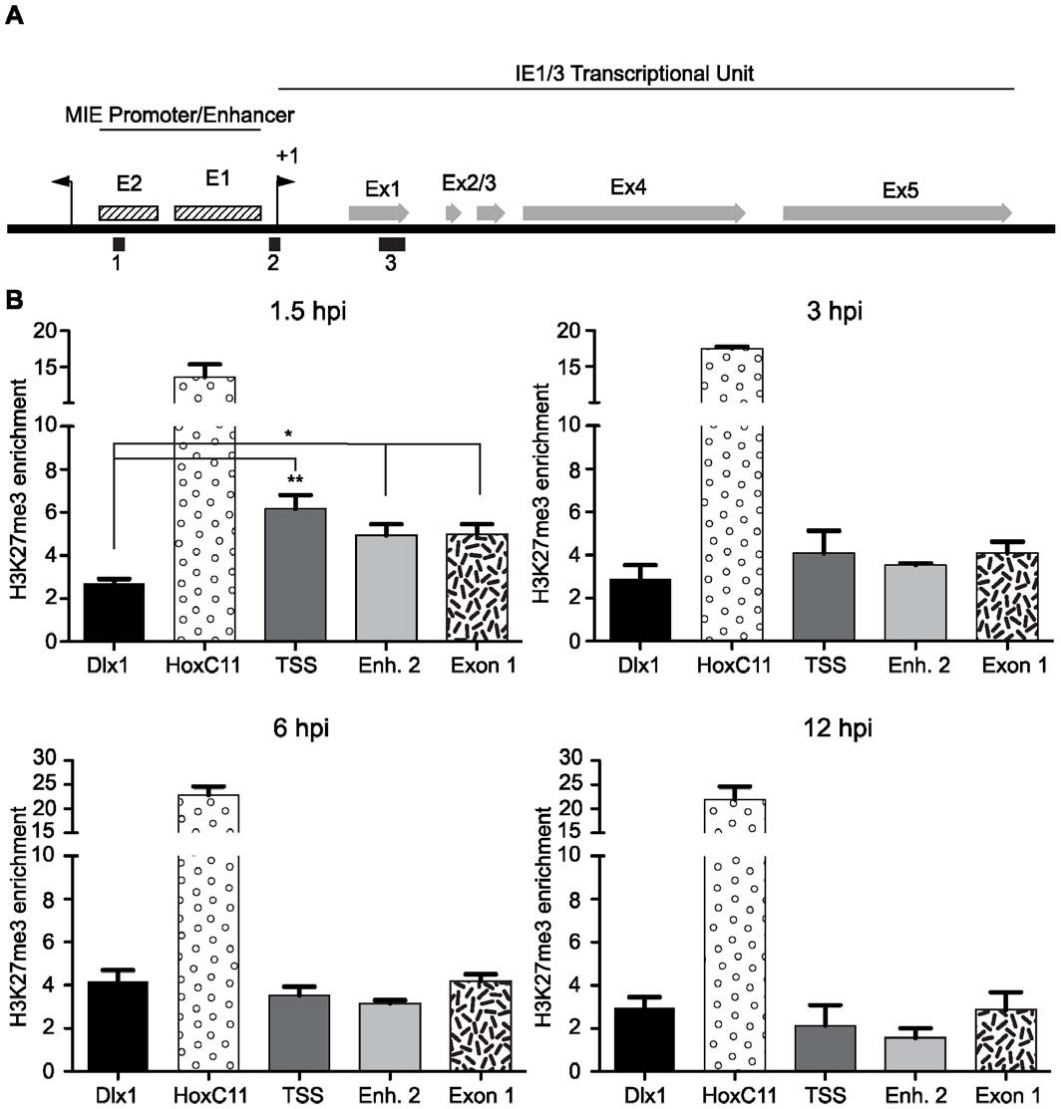
Measurement of H3K27me3 enrichment at the MIE locus of MCMV. (A) Graphical illustration of the MIE locus of MCMV. Solid black boxes represent regions probed for H3K27me3 & H3K4me3 enrichment. Regions denoted in figure: (1) Represents the Enhancer 2 region, (2) the transcriptional start site (TSS) of the IE1-3 transcriptional unit and (3) represents Exon 1 within the ORF of the IE1-3 locus. +1 transcriptional start sites are indicated with black arrows. Enhancer regions are marked in hatched boxes. IE1-3 exons are depicted as gray arrows. (B) H3K27me3 is enriched at the MIE locus at pre-IE times during MCMV infection. ChIPs using anti-H3K27me3 antibody were analyzed by Q-PCR using primer/probe sets specific for the indicated loci at 1.5, 3, 6 and 12 hpi as indicated. Each graph displays the mean value and S.E.M. for each queried locus, with data from three independent ChIPs. The results are presented as the IgG subtracted %Input of the queried locus normalized to the IgG subtracted β-Actin %Input. Samples with values that vary significantly from the negative control, Dlx1,are indicated by asterisks (* *P* value<0.05, ** *P* value<0.01; ANOVA).

**Figure 3:**
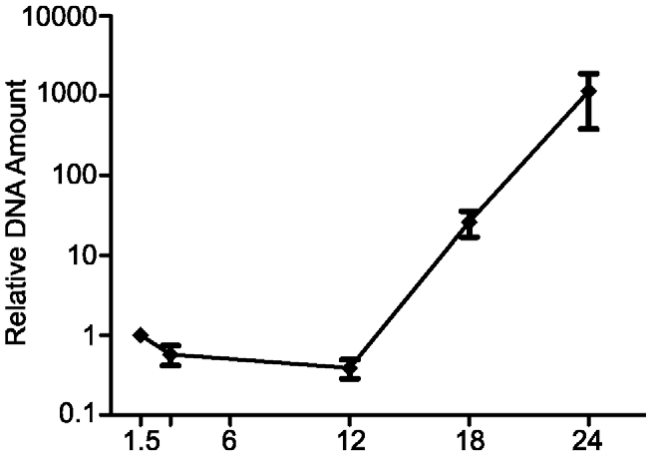
Viral DNA replication kinetics in mouse fibroblasts. MCMV DNA content on total DNA collected from MCMV infected cells analyzed by Q-PCR at 1.5, 3, 12, 18 and 24 hpi as indicated. Each graph displays the mean value and S.E.M. for, with data from three independent assays. The results are presented as the relative IE1-3 DNA amount normalized to a cellular reference, Dlx1.

Fold-enrichment of the H3K27me3 mark at selected loci within the Major Immediate-Early (MIE) locus and cellular genes was measured by Taqman quantitative PCR (Q-PCR) analysis relative to β-actin. ChIP assays were performed on chromatin prepared from mouse fibroblasts infected with MCMV at a multiplicity of 1.0 and harvested at 1.5, 3, 6 and 12 hours post-infection (hpi). Using Q-PCR, we probed three loci within the MCMV MIE region for the presence of H3K27me3: (1) the transcriptional start site (TSS), (2) the Enhancer 2 element and (3) Exon 1 (Fig. 2A) and examined the enrichment for significance relative to the negative control, Dlx1. At 1.5 hpi, we observed significant enrichment of H3K27me3 at the TSS (6.2-fold), Enhancer 2 (4.9-fold) and Exon 1 (5.0-fold) that was well above the 2.7-fold enrichment observed at the negative control Dlx1 (Fig. 2B). The significant enrichment of H3K27me3 is rapidly lost from the TSS, Enhancer 2 and IE1-3 exon1 at 3 hpi, even though enrichment at the negative control gene, Dlx1 remains constant from 1.5–12 hpi. Over the course of infection, we also noticed a significant increase in H2K27me3 at the HoxC11 promoter by 6 hpi, suggesting infection may impact cellular targets of PRC2 (Fig. S1A). We also performed ChIPs to measure fold-enrichment of total histone H3 at these loci. H3 levels at the TSS, Enhancer 2 and IE1-3 exon1 remained constant at the 1.5 and 3 hour time points, suggesting that the loss of H3K27me3 at these loci is not a consequence of the loss of bulk histone H3 (Fig. S1B) . It is also possible that the loss of H3K27me3 on the viral genome was due to amplification of viral copy number, effectively diluting the number of genomes bound by H3K27me3. However, by Q-PCR analysis, we detected no appreciable increase in viral genome copy number until after 12 hpi (Fig. 3). In summary, our data indicate that H3K27me3 is significantly enriched at the MCMV MIE locus at pre-immediate early times of infection, suggesting the cellular complex PRC2 targets MCMV genomes for heterochromatin formation. However, this repressive chromatin structure is overcome, as indicated by the rapid loss of H3K27me3 from the MIE locus as the viral replication cycle proceeds.

### H3K4me3 is enriched at the MCMV MIE locus at immediate-early times of infection

Histone H3 trimethylation on lysine-4 (H3K4me3) is strongly associated with transcriptional activation [13]. To determine if MCMV genomes become associated with H3K4me3 during the IE phase of infection, we again performed ChIP analysis of chromatin prepared from mouse fibroblasts infected with wild-type MCMV. To demonstrate that our immunoprecipitations were specific, we again validated our ChIP samples by assessing the enrichment of H3K4me3 at HoxC11, Dlx1 and β-actin. As a transcriptionally active gene, Dlx1 is the positive control, as it is known to be enriched in H3K4me3, whereas, the transcriptionally silent HoxC11 gene is devoid of this mark [32]. We observed a 34.7-fold enrichment of H3K4me3 at the promoter of Dlx1 over the negative control, HoxC11 (*P* value<0.001; ANOVA) (Fig. 1B).

**Figure 4:**
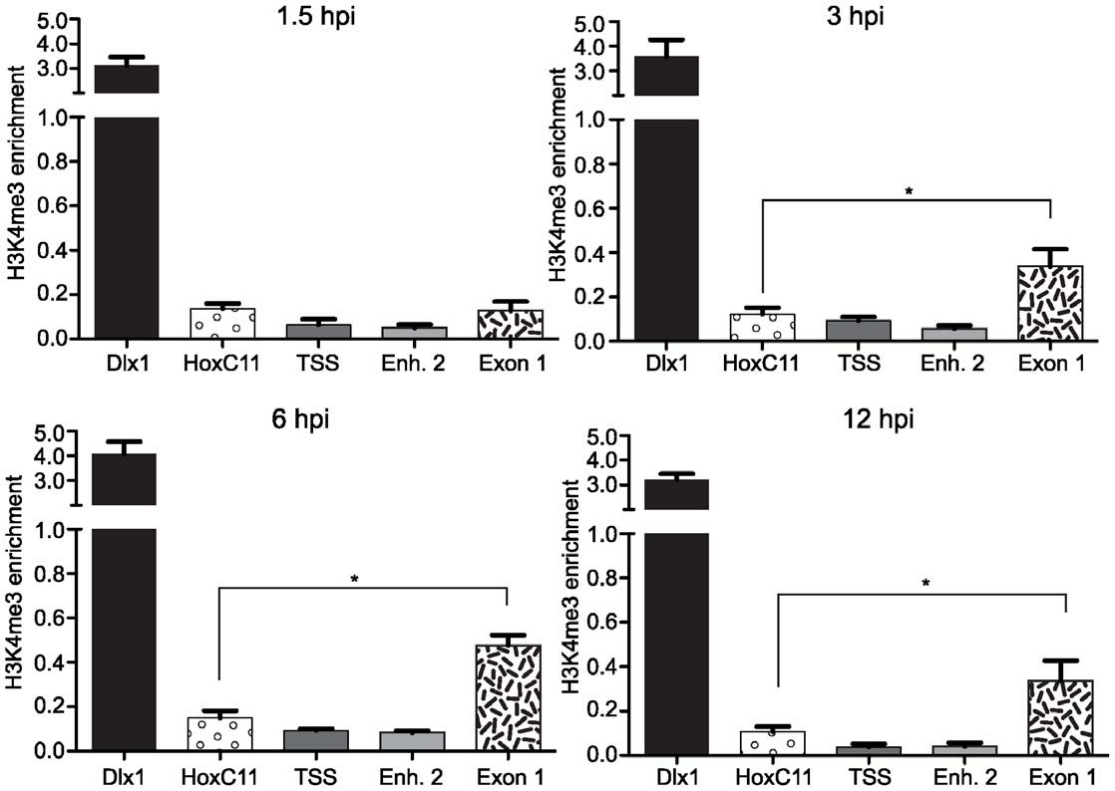
Measurement of H3K4me3 enrichment at the MIE locus of MCMV. ChIPs using anti-H3K4me3 antibody were analyzed by Q-PCR using primer/probe sets specific for the indicated loci at 1.5, 3, 6 and 12 hpi as indicated. Each graph displays the mean value and S.E.M. for each queried locus, with data from three independent ChIPs. The results are presented as the IgG subtracted %Input of the queried locus normalized to the IgG subtracted β-Actin %Input. Samples with values that vary significantly from the negative control, HoxC11, are indicated by asterisks (* *P* value<0.05; ANOVA).

Fold-enrichment of the H3K4me3 mark at selected loci within the MIE locus relative to β-actin was measured by Taqman Q-PCR analysis. ChIP assays were performed on chromatin prepared from mouse fibroblasts infected with MCMV at 1.5 to 12 hpi with antibody specific for H3K4me3. Relative occupancy of H3K4me3 at the MIE is detectable but less than our reference gene β-actin. Hence, the fold-enrichment of H3K4me3 at the MIE locus has a value<1, but is nevertheless meaningful. At 3, 6 and 12 hpi, we observed significant enrichment (0.34, 0.48, 0.34-fold enrichment, respectively) of H3K4me3 at Exon 1, relative to β-actin, that was well above the 0.1-fold enrichment observed at the negative control HoxC11 (Fig. 4). We detected no significant enrichment of H3K4me3 at the TSS or Enhancer 2 loci at any of the time points analyzed. In summary, our data indicate that H3K4me3 is significantly enriched at a region within the MIE locus, and remains so, throughout the IE period.

### MCMV infection alters the nuclear staining of PRC2 members

**Figure 5:**
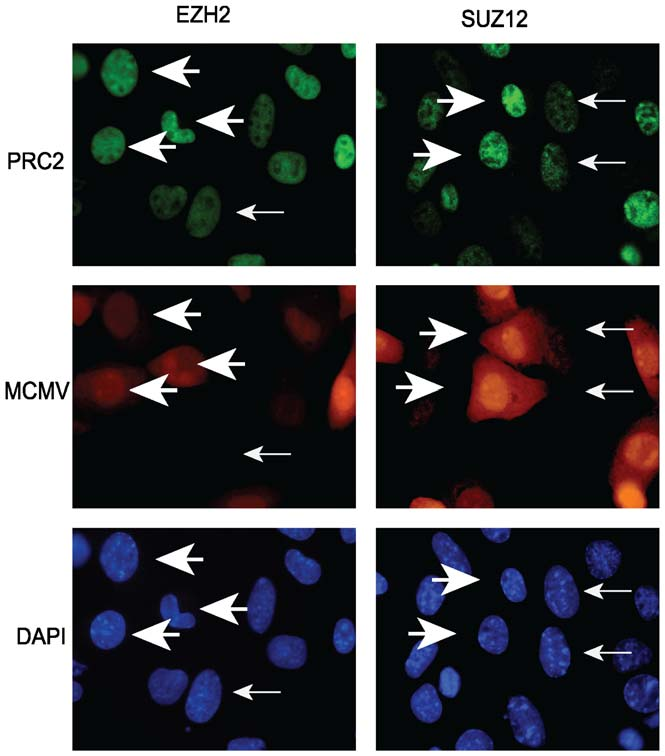
Immunofluorescence assay for PRC2 proteins during MCMV infection. At 12 hpi, mock-infected or MCMV:mCherry-infected fibroblasts were fixed and probed with antibodies against the PRC2 components, EZH2 or SUZ12. Large arrowheads indicate MCMV infected cells that also exhibit increased immunofluorescence signal for EZH2 or SUZ12, while small arrows indicate uninfected neighboring cells. All images are at 40× magnification.

The rapid decrease in H3K27me3 at the MIE locus from 1.5 to 3 hpi suggested that MCMV infection may specifically antagonize the deposition of this mark on viral chromatin. Therefore, we investigated the consequences of MCMV infection on the sub-cellular localization of selected PcG proteins as well as PcG protein expression levels. We first performed immunofluorescence assays to monitor the sub-cellular localization and distribution of PRC2 proteins in response to MCMV infection. Mouse fibroblasts were infected with MCMV at an MOI of 1.0. We used recombinant MCMV that expresses the fluorescent mCherry protein under the control of the SV40 promoter to permit easy identification of MCMV-infected cells. At 12 hpi, cells were fixed and stained with antibodies specific for the PRC2 proteins EZH2 and SUZ12. As shown in Figure 5, we observed a marked increase in both Suz12 and Ezh2 staining in MCMV infected cells compared to uninfected cells.

**Figure 6:**
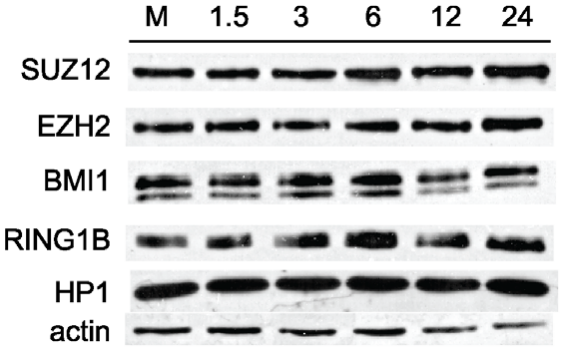
Western blot analysis of PcG protein expression during MCMV infection. Whole cell lysates were prepared from mock-infected or MCMV-infected fibroblasts at the indicated times. Blots were probed with antibodies to the indicated PcG proteins as well as antibodies against HP-1 and actin as loading controls.

To determine whether the observed, infection-dependent, increase in PcG immunofluorescence could be explained by an overall increase in protein expression, we next performed western blot analysis for the PRC2 proteins EZH2 and SUZ12 as well as the PRC1 proteins BMI1 and RING1B (Fig. 6). Mouse fibroblasts were infected with MCMV and harvested from 1.5 hpi to 24 hpi. Whole cell lysates were prepared and PcG protein levels were assessed. Compared to mock-infected cells, EZH2, SUZ12, BMI1 and RING1B proteins levels were largely unchanged throughout the course of the experiment. These results suggest that MCMV infection has little global effect on the PcG protein expression levels.

### MCMV stimulates PRC2 association with chromatin during the early phase of lytic infection

**Figure 7:**
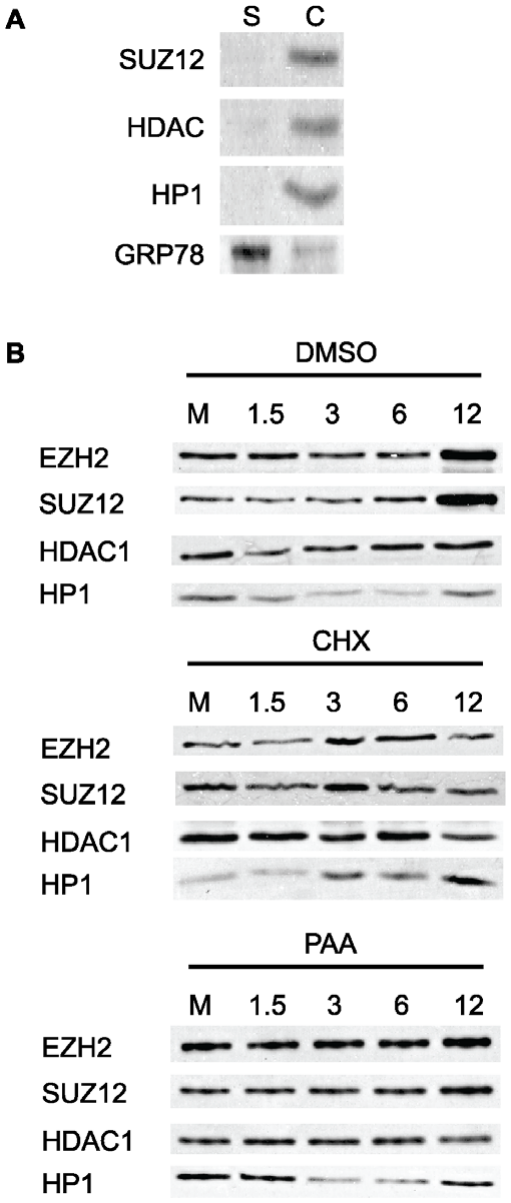
Western blot analysis of PRC2 association with chromatin during MCMV infection. (A) Validation of chromatin fractionations. Mouse fibroblasts were fractionated as described in the Materials and Methods. 25 µg of protein from the soluble (S) and chromatin (C) fractions were probed for the chromatin-associated proteins SUZ12, HDAC1 and HP1. The blot was then stripped and re-probed for the ER-associated protein GRP78. (B) Chromatin fractions prepared from vehicle-treated (DMSO), cycloheximide (100 µg/ml) or phosphonoacetic acid (200 µg/ml) treated, mock-infected (M) or MCMV-infected fibroblasts at the indicated times. Blots were probed with antibodies against the PRC2 components EZH2 and SUZ12, or HDAC1 and HP1 for a loading control and fractionation quality control, respectively.

We hypothesized that the increase in PRC2-staining in MCMV infected cells observed in our immunofluorescence assays reflects an increased association of PRC2 with chromatin during infection. To test this hypothesis, we performed chromatin fractionation experiments designed to assay changes in the amount of salt-sensitive, chromatin-bound EZH2 and SUZ12 in mock-infected and MCMV-infected mouse fibroblasts (MOI 5) from 1.5 hpi to 12 hpi. We also monitored HP1 and HDAC1 levels as reliable indicators of fractionation efficiency and protein loading, respectively. As shown in Figure 7, between 6 and 12 hpi we observed a substantial increase in both EZH2 and SUZ12 association with chromatin, compared to mock-infected cells. This data suggests that the increase in SUZ12 and EZH2 staining-intensity in the IFA in response to MCMV infection is likely due to the increased nucleation of PRC2 complexes, from the nucleoplasm, onto chromatin.

To determine whether viral gene expression was necessary for PRC2 enrichment on chromatin, we performed chromatin fractionations on mock or MCMV-infected cells in the presence of cycloheximide (Fig. 7). Treatment with cycloheximide prevented the accumulation of both EZH2 and SUZ12 in the chromatin fraction over the course of the experiment. This data suggests that viral gene expression is required for the increase in PRC2 association with chromatin we observe during infection. To determine if viral DNA replication was necessary for PRC2 enrichment on chromatin, we next performed chromatin fractionations on mock- or MCMV-infected cells treated with phosphonoacetic acid (PAA). Inhibition of viral DNA replication by PAA, led to a minimal accumulation of EZH2 and Suz12 in the chromatin fraction (Fig. 7). This data indicates that viral DNA replication is also a required step in the increased association of PRC2 with chromatin we observed during MCMV infection.

### H3K27me3 levels increase during MCMV infection

**Figure 8:**
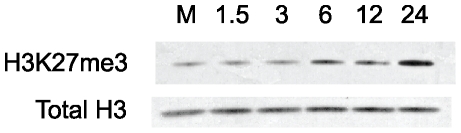
Western blot analysis of cellular H3K27me3 levels during MCMV infection. Whole cell lysates were prepared from mock-infected or MCMV-infected fibroblasts at the indicated times. Blots were probed with antibodies against H3K27me3 or total H3.

Since MCMV infection enhanced PRC2 association with chromatin, we hypothesized that H3K27me3 levels would also increase during infection. To test this hypothesis, we performed Western blot analysis for H3K27me3 on whole cell lysates prepared from mock- and MCMV-infected cells (MOI 5). Compared to mock-infected cells, we detected an increase in the amount of H3K27me3 in MCMV infected cells at 6 hpi that continued to increase between 12 and 24 hpi (Fig. 8).

### PRC2 localizes to MCMV replication compartments during lytic infection

**Figure 9:**
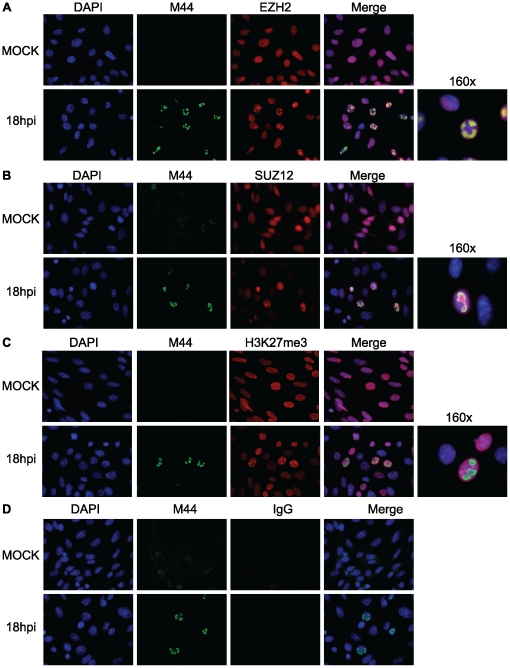
Co-immunofluorescence assay for PRC2 enrichment within MCMV replication compartments. At 18 hpi, mock-infected or MCMV-infected fibroblasts were fixed and incubated with antibodies against M44 and (A) EZH2, (B) SUZ12 or (C) H3K27me3. (D) Purified rabbit IgG served as an isotype control. The first column of panels displays DAPI staining for nuclei. The second column of panels displays M44 staining, marking MCMV replication compartments. The third column of panels displays staining EZH2, SUZ12, H3K27me3 or isotype IgG. The fourth column of panels displays a merged image of the first three channels. The fifth column of panels displays a high-magnification image of the merged image from the fourth column. All images are 40×, unless otherwise indicated.

Since we found that PRC2 chromatin-association required viral DNA replication, we asked if PRC2 could be found in viral replication centers as infection progressed. We performed co-immunofluorescence assays on MCMV-infected fibroblasts, to detect the DNA polymerase accessory protein M44, a marker for replication compartments, in combination with EZH2, SUZ12 or H3K27me3. At 18 hpi we were able to detect enrichment of EZH2 and SUZ12 within MCMV replication compartments, as defined by M44 staining (Fig. 9A and B). We observed this pattern as early as 12 hpi, and as late as 24 hpi (Figs. S2 and S3). Surprisingly, although total nuclear H3K27me3 protein increased as measured by western blot (Fig. 8) and nuclear H3K27me3 immunofluorescence appeared to increase in infected cells, MCMV replication compartments were relatively deficient for this specific histone PTM (Fig. 9D). The staining patterns for all three antibodies used separately, were identical to that observed when used in combination, suggesting our co-immunofluorescence assays represent a true enrichment of both EZH2 and SUZ12 within viral replication compartments. In summary, our results demonstrate recruitment of PRC2 to the MCMV replication compartment as early as 12 hpi, with enrichment increasing as the viral replication cycle proceeds. However, H3K27me3 was dramatically under-represented within the replication compartment, suggesting PRC2 enzymatic activity on newly replicated viral genomes may be inhibited.

## Discussion

The assembly of chromatin on viral DNA plays a significant role in the execution of the lytic or latent replication program of herpesviruses. During lytic replication, viral genomes initially associate with nucleosomes with an irregular distribution over the genome and at levels well below that of cellular genes. As replication proceeds, newly synthesized viral genomes become increasingly associated with nucleosomes. Since herpesvirus genomes acquire modifications typical of transcriptionally active euchromatin as infection proceeds, it is generally thought that chromatin structure can play a significant regulatory role in lytic gene expression. Several studies have implicated the formation of heterochromatin on cytomegalovirus genomes as an important step in both lytic and latent infections, using *in vitro* and *in vivo* model systems [23], [25], [33], [34]. However, the molecular mechanisms governing the establishment of heterochromatin and its regulatory role during infection remain poorly understood.

In our study, we performed a detailed analysis of the association of H3K27me3, the central marker of facultative chromatin, with the MIE locus of the MCMV genome. We observed that H3K27me3 is rapidly deposited on the viral genome after it enters the nucleus, perhaps as a cellular response to the introduction of foreign DNA or perhaps due to intrinsic sequence-specific signals within the MIE locus that promote the formation of a heterochromatin-like structure. However, the enrichment of H3K27me3 is short-lived, as the immediate-early phase of replication proceeds H3K27me3-enrichment declines, and we observed a concomitant increase in H3K4me3-enrichment at Exon 1 within the MIE locus. H3K4me3 is catalyzed by the mammalian Trithorax complex and may promote transcriptional activation by recruiting ATP-dependent chromatin remodelers and stimulating transcriptional elongation [13]. Our observations suggest that the dynamics of H3K27me3- and H3K4me3-enrichment at the MIEP of MCMV are much like that of PcG-repressed cellular genes that become activated in response to various stimuli. Although we have yet to investigate the mechanism of PcG and TrxG recruitment to the MIE locus, it may likely involve transcription factors that bind to the promoter/enhancer region. Analysis of the MCMV MIEP by another group revealed numerous potential binding sites for the transcription factor YY1, a PRC-interacting protein that possesses both repressing and activating functions [24]. Robust recruitment of this protein to the MIEP was observed during the acute and latent phases of infection and could promote PRC interactions with the MIEP. Alternatively, enrichment of fHC or EC at the MIEP may be dependent on transcription factors functioning differentially in a cell-type specific manner.

In an effort to elucidate the consequences of MCMV infection on PRC2 function and assembly, we discovered that MCMV infection results in an increased association of EZH2 and SUZ12 with chromatin. Although it is possible that the increased detection of both EZH2 and SUZ12 in infected cells represents increases in epitope availability, perhaps due to changes in PRC2 complex composition or stability, we propose the observed fluorescence increase represents the nucleation of PRC2 onto chromatin, as supported by our chromatin fractionation data. The mechanism that recruits PRC2 into viral replication centers is currently unknown; viral perturbation of the cell-cycle or a novel viral determinant could promote the enrichment of PRC2 proteins in viral replication centers. Interestingly, both cycloheximide and PAA treatment of MCMV-infected fibroblasts prevented the observed increase in association of both SUZ12 and EZH2 with chromatin seen in untreated, MCMV-infected cells. This comparative analysis suggests the association of PRC2 with chromatin is dependent on the formation and maturation of the viral replication compartment, including DNA replication. Strengthening the physical link between viral DNA replication and PRC2, we observed a distinct enrichment for both EZH2 and SUZ12 within the replication compartment. The association of PRC2 with chromatin during viral DNA replication suggests PRC2 may be recruited to viral replication centers. Indeed, in human fibroblasts, PRC2 co-localizes with PCNA at DNA replication forks during S-phase [35].

Our observation of PRC2 enrichment within the replication compartment suggested that H3K27me3 could be associated with newly replicated viral genomes. Within the cytomegalovirus replication compartment, newly replicated viral DNA associates with core histones generating nucleosomal frequencies, at some loci, that approach those observed for host chromatin [21]. Other studies have shown that histone PTMs provide regulatory functions within the replication compartment, as histone PTMs associated with transcriptional activation are found on late promoters, at late times post infection [22]. Surprisingly, though, our results indicate PRC2 activity within the viral replication compartment may be blocked, as the H3K27me3 enrichment contained within these structures was, in fact, minimal based on our IFAs. Formally, we may lose our ability to detect H3K27me3 within viral replication centers; however, we can detect H3K4me3 within the replication compartment (data not shown), suggesting at least a portion of the H3 N-terminal tail is immunologically reactive. Productive CMV infection hijacks several cellular processes, namely cell cycle regulation and major signaling pathways that impact PRC2 stability, substrate affinity and enzymatic activity [36], [37], [38], [39]. Consequently, PRC2 targeted to viral replication compartments, may be functionally impaired. Alternatively, recruited PRC2 may provide a function independent of histone H3 post-translational modification. A third explanation could be that CMV replication forks may contain or recruit cellular antagonists of PRC2, thereby uncoupling PRC2 activity from newly synthesized genomes [40]. Recruitment of the H3K27-specific demethylases KDM6A and KDM6B to viral DNA replication machinery could, in fact, counteract faithful transmission of PRC2 catalyzed heterochromatic PTMs to daughter DNA strands. Thus, the activity of these demethylases early at the onset of viral DNA replication would allow for rapid expansion of viral chromatin lacking PRC2-catalyzed heterochromatin. Further, strong evidence exists for an interaction between KDM6A/B and the Trithorax complex, potentially creating a euchromatic chromatin environment refractory for H3K27me3 deposition [41], [42].

Paradoxically, we observed that total H3K27me3 levels increase over time in infected fibroblasts, and our ChIP data revealed a significant increase in H3K27me3 enrichment at the HoxC11 promoter over the course of infection. Collectively, these observations provide evidence for lytic infection affecting host chromatin dynamics. We would predict, in infected cells, H3K27me3 enrichment at cellular chromatin would be enhanced, at numerous PRC2 responsive regions. We postulate that host chromatin increases in H3K27me3 levels may occur as a consequence of virally induced nuclear reorganization, including the exclusion of host chromatin to the nuclear margins in infected cells, allowing promiscuous transfer of epigenetic information between adjacent strands of host DNA that would otherwise be well separated within the nucleus. Alternatively, functional antagonists of PRC2 activity, such as KDM6A and KDM6B, may be actively recruited to viral replication compartments, leaving PRC2 activity at cellular chromatin unchecked.

It is likely that cytomegalovirus has evolved mechanisms to interfere with the inhibitory chromatin structure and promote an active conformation on viral promoters for lytic replication to proceed efficiently. To prevent sustained inhibition by the cellular protein complexes that establish and maintain heterochromatin, we postulate there must be viral determinants that promote the removal of H3K27me3 from the viral genome, prevent continued methylation of H3K27 and recruit cellular proteins that modify chromatin structure to permit robust transcriptional activation. It is well established that tegument and IE proteins antagonize the activity of histone deacetylases and interact with protein complexes that modify and remodel nucleosomes to facilitate transcriptional initiation and elongation by RNA polymerase II [43], [44], [45]. Future studies will aim to identify viral determinants modulating, directly or indirectly, PcG mediated epigenetic repression. Further, it will be important to identify the scope with which PRCs influence cytomegalovirus gene expression during lytic and latent types of infection. Polycomb mediated transcriptional repression is plastic in nature, and represents a robust cellular programming system capable of executing gene expression patterns in an exact manner. Similarly, the transcriptional programs for both lytic and latent cytomegalovirus infections are dynamic, and executed in a highly regulated fashion. A controlled interaction between Polycomb Repressive Complexes and CMV may confer advantageous properties for the virus, by regulating cell- and context-dependent transcriptional programs.

## Materials and Methods

### Cells and virus

10.1 mouse embryo fibroblasts [46] were propagated in DMEM with 10% fetal calf serum. Wild-type MCMV was derived from pSM3fr, a BAC clone of the Smith strain and titered by plaque assay on 10.1 cells [47], [48].

Two wild-type recombinant derivatives of MCMV were used in experiments. MCMV:mCherry expresses the fluorescent mCherry protein under the control of the SV40 promoter. The SV40-mCherry cassette is inserted at the m156 ORF and this recombinant virus grows with wild-type kinetics in fibroblasts. MCMV:m106-GFP is a recombinant MCMV that expresses the m106 ORF as a green fluorescent protein fusion. This recombinant virus expresses m106-GFP at late times of infection and grows with wild-type kinetics in fibroblasts (manuscript in preparation). Virus was inactivated by UV treatment using a Stratalinker 1800 (Stratagene) and exposing the virus to 2.4×10^6^ μJoules of UV radiation. Virus inactivation was assessed by plaque assay.

### Primers

Quantitative PCR (Q-PCR) reagents and Taqman probes were purchased from Applied Biosystems. Taqman assays were designed using MacVector or Primer-BLAST (http://www.ncbi.nlm.nih.gov/tools/primer-blast/). A full listing of primer/probe sequences used in our studies is found in Table 1.

**Table 1:** Q-PCR Primers and Probes.

Dlx1	Fwd:	ACACCCAGACCCCGCGAGAA
	Rev:	ACACAGCCTTGCCCGACAC
	Probe:	TGACCATGACCACCATGCCAGA
HoxC11	Fwd:	GGAACCGTCCGGGAAGTGGC
	Rev:	TGCATAAGCTCGTCGGCCGC
	Probe:	ACCATCGGAACAGCTACTCGTCT
β-Actin [23]	Fwd:	CGTTCCGAAAGTTGCCTTTTA
	Rev:	GCCGCCGGGTTTTATAGG
	Probe:	CTCGAGTGGCCGCTG
MIE TSS [23]	Fwd:	GGTGGTCAGACCGAAGACT
	Rev:	GCTGAGCTGCGTTCTACGT
	Probe:	CTGGTCGCGCCTCTTA
MIE Enhancer 2	Fwd:	TTTGACTCAGTGTGCTTGCC
	Rev:	TGACTCAATGGCCTTTACCC
	Probe:	TTGGCTTACCTCCCATTGAC
MIE Exon 1	Fwd:	TTCGCAGTACGAGCAACAAC
	Rev:	TGCTCAGATCGTCTCCTGTG
	Probe:	CAGGAACTGACGGAGTCCTC

### Antibodies

Antibody sources and usage for different experiments are as follows: EZH2 (WB 1∶1000, IF 1∶200), SUZ12 (WB1∶1000, IF 1∶800) and H3K4me3 (ChIP 5 ug) were purchased from Cell Signaling. BMI1 (WB 1∶2500), RING1B (WB 1∶2500), pan-H3 (WB 1∶5000) and H3K27me3 (ChIP 5 ug, IF 1∶200, WB 1∶5000) were purchased from Millipore. HDAC1 (WB 1∶1000), HP1 (WB 1∶1000), and GRP78 (WB 1∶1000) were purchased from Santa Cruz. β-Actin (WB 1∶15,000), Goat anti-Mouse/Alexa 488 (IF 1∶1000) and Goat anti-Mouse/Alexa 594 (IF 1∶1000) were purchased from Invitrogen. Goat anti-Rabbit/FITC (IF 1∶200), Goat anti-Rabbit/HRP (WB 1∶10,000) and Goat anti-Mouse/HRP (WB 1∶10,000) were purchased from Jackson Labs. The M44 (IF 1∶500) antibody was a kind gift from Dr. Carol Wu at the University of Connecticut Health Sciences Center. Normal Rabbit IgG (ChIP 5 ug) was purchased from Abcam.

### SDS-PAGE

Mock-infected and MCMV-infected cells were scraped and collected in PBS; following two PBS washes, cells were centrifuged and either flash frozen at −80°C for later use or processed for SDS-PAGE. Cells were lysed in RIPA buffer (150 mM NaCl, 1% v/v Nonidet P-40, 0.5% w/v deoxycholate, 0.1% w/v SDS, 5 mM EDTA, 50 mM Tris; pH 8.0) containing protease inhibitor cocktail (ThermoScientific). The cell lysate was briefly sonicated to facilitate nuclear protein release and insoluble debris was centrifuged. The soluble lysate was assayed for protein content using Bradford reagent (Bio-Rad). 50 µg of protein from each sample was separated by SDS-PAGE on a 12% gel.

### Western Blotting

Following transfer of SDS-PAGE-separated proteins to nitrocellulose, blots were blocked in 5% dry milk in PBST or TBST, depending on manufacturer recommendations for each antibody, for 1–2 hours at room temperature. Blots were incubated with primary antibody diluted in blocking buffer overnight at 4°C. After washing, blots were incubated with secondary antibody diluted 0.1% dry milk in PBST or TBST for 1 hour at room temperature. Chemiluminescent detection was performed using SuperSignal West Pico Chemiluminescent Substrate (ThermoScientific).

### Chromatin Fractionation

Separation of salt sensitive chromatin binding proteins from soluble proteins was performed as described in [49]. Briefly, fibroblasts were infected with MCMV at an MOI of 1.0 or mock-infected. 5×10^6^ cells were rinsed and resuspended in 1 ml cold PBS. Following centrifugation, the cell pellet was resuspended in NB buffer (15 mM Tris pH7.4, 0.3% NP40, 5 mM MgCl_2_, 60 mM KCl, 15 mM NaCl, 1 mM DTT, 250 mM Sucrose) and incubated on ice for 5 minutes. Following centrifugation at 1,300×g the supernatant (soluble fraction) was collected. The pellet was rinsed once in NB and extracted in TTEM-250 (20 mM Tris pH7.4, 1% Triton X-100, 2 mM EDTA, 10 mM MgCl_2_, 250 mM NaCl, 10% Glycerol) and incubated on ice for 30 minutes. Following centrifugation at 10,000×g the supernatant (chromatin fraction) was collected and analyzed by western blot for EZH2, SUZ12, HDAC1 and HP1 proteins.

### Immunofluorescence

Mouse fibroblasts were plated on sterile glass cover slips for immunofluorescence assays. At the time of the assay, mock-infected and MCMV-infected cells were washed with PBS and then fixed with 4% paraformaldehyde in ddH_2_0, pH 7.4 for 15 minutes, or fixed in methanol at −20°C for 10 min. Post fixation, free aldehyde groups were quenched using ice cold 0.1 M glycine in PBS. Cells were permeablized on ice by incubation in cold permeablization buffer (0.25% Triton X-100 in PBS) for 10 minutes. Cells were then washed 3 times for 5 minutes each in cold PBST (0.2% Tween 20 in PBS) followed by incubation with blocking buffer (5% BSA, 0.2% Tween20 in PBS) for 1–2 hours at room temperature. Primary antibody was diluted in blocking buffer and incubated with the cells in a humid chamber for 1–2 hours at 37°C. Cover slips were then washed 3 times 5 minutes each in cold PBST. Secondary antibody incubations and washes were performed as described for primary antibody. Following the last PBST wash, cover slips were rinsed 2 times in ice cold PBS and then mounted onto glass microscope slides using SlowFade Reagent (Invitrogen) containing DAPI.

### Image Acquisition

All imaging was performed in the University of Colorado Denver Advanced Microscopy Core Facility. Confocal image acquisition was done with an Olympus IX81 inverted motorized microscope with spinning disk attachment. UAPO 40× oil N.A. 1.35, and U PLAN APO 100× oil N.A. 1.35 objectives were used. Excitation source was from a 100 watt Hg lamp. Image collection was completed with a Hamamatsu ORCA IIER monochromatic CCD camera 1344×1024 full chip, 6.45×6.45 µm pixel. Filter sets used were, for DAPI: Excitation 360-70 & Emission 420-60, for FITC: Excitation 450-80 & Emission 535 and for TRITC Excitation 535 & Emission 635. Intelligent Imaging Slidebook version 4.067 was used as acquisition software. Immunofluorescence data was collected from many fields of view across multiple independent experiments. Collected images were processed using Image J software (NIH).

### Chromatin Immunoprecipitation (ChIP)

1×10^7^ mock-infected or MCMV-infected fibroblasts were fixed with 1% formaldehyde/PBS for 10 minutes at room temperature. Fixation was terminated by adding glycine/PBS to a final concentration of 125 mM and incubating for 5 minutes at room temperature with rocking. Cells were scraped in DMEM containing 10% Newborn Calf Serum and protease inhibitor cocktail (Roche) and collected by centrifugation at 800× *g* for 5 minutes. The cell pellet was resuspended and washed twice with ice cold PBS containing protease inhibitor cocktail. Cells were lysed in 1 ml IP Lysis Buffer (150 mM NaCl, 0.5% Nonidet P-40, 1% Triton X-100, 5 mM EDTA, 50 mM Tris pH 7.5) for 10 minutes and the nuclei collected by centrifugation for 5 minutes at 12,000× *g*. Nuclei were resuspended in Nuclear Lysis Buffer (1% SDS, 10 mM EDTA, 50 mM Tris pH 8.1) and mechanically disrupted with several passages through a 22G1 syringe needle. Chromatin was sonicated to fragments 200–1000 bp in length using a Fisher 100 Sonic Dismembrator at a power setting of 8. Sonication was performed as 8 pulses of 5 seconds each with 2 minutes of rest on ice between each pulse. Insoluble aggregates were cleared by centrifugation at 14,000× *g* for 10 min. For each immunoprecipitation (IP), ∼2×10^6^ cell equivalents of cleared chromatin was diluted 1∶10 in ChIP dilution buffer (167 mM NaCl, 0.01% SDS, 1.1% Triton X-100, 16.7 mM Tris pH 8.1, 1 mM EDTA) containing protease inhibitor cocktail supplemented with 1 mM PMSF. Antibody (5 µg) and Protein A-coupled magnetic beads (20 µL) (Millipore), were added to each IP and incubated at 4°C overnight with rotation. Magnetic beads retaining the immune complexes were then washed. After the final wash, beads were resuspended in 100 µl 10% Chelex 100 resin (BioRad) and 40 µg Proteinase K. After brief vortexing, samples were incubated at 57°C for 45 minutes on a Thermomixer (Eppendorf) with shaking. Samples were then incubated at 96°C for 20 minutes on a Thermomixer with shaking, then cooled on ice and collected by centrifugation. The supernatant was collected and assayed by quantitative PCR. 1% of each of the input chromatin DNA and the ChIP DNA samples were assayed by Q-PCR in triplicate using a Roche LightCycler 480. Fold-enrichments of histone marks at various genomic loci were calculated as IgG-subtracted %Input of the queried locus divided by the IgG-subtracted %Input of the reference gene β-actin. Statistical comparisons were made by one-way ANOVA followed by Tukey's multiple comparisons post-test using Graph Pad Prism 5 software.

### Viral DNA Replication Assay

Mouse fibroblasts were infected with WT MCMV:mCherry at a MOI of 1. At 1.5, 3, 12, 18 and 24 hpi cells were harvested by scraping, washed 2 times in PBS and then flash frozen until use. For total DNA collection, samples were processed using a QIAamp DNA Mini Kit (Qiagen) according to the manufacturer's recommendations. Viral DNA was quantified by Q-PCR in triplicate, using the IE1-3 primer set. Each sample was normalized to cellular Dlx1 DNA levels.

## Supporting Information

Figure S1 **ChIP analysis of (A) H3K27me3 enrichment at the HoxC11 locus and (B) histone H3 at the MIE locus during MCMV infection.** H3K27me3 enrichment was measured in mouse fibroblasts from 1.5 to 12 hpi. (* *P* value<0.05, ANOVA). ChIPs using anti-H3 antibody were analyzed by Q-PCR using primer/probe sets specific for the indicated loci at 1.5 (black bars) and 3 hpi (gray bars) as indicated. Each graph displays the mean value and S.E.M. for each queried locus, with data from two independent ChIPs. The results are presented as the IgG subtracted %Input of the queried locus normalized to the IgG subtracted β-Actin %Input.
(TIF)

Figure S2 **Co-immunofluorescence assay for PRC2 enrichment within MCMV replication compartments.** At 12 and 24 hpi, mock-infected or MCMV-infected fibroblasts were fixed and incubated with antibodies against M44 and (A) EZH2 or (B) SUZ12. The first column of panels displays DAPI staining for nuclei. The second column of panels displays M44 staining, marking MCMV replication compartments. The third column of panels displays staining EZH2 or SUZ12. The fourth column of panels displays a merged image of the first three channels. The fifth column of panels displays a high-magnification image of the merged image from the fourth column. All images are 40×, unless otherwise indicated.
(TIF)

Figure S3 **Co-immunofluorescence assay for PRC2 enrichment within MCMV replication compartments.** At 12 and 24 hpi, mock-infected or MCMV-infected fibroblasts were fixed and incubated with antibodies against M44 and (A) H3K27me3 or (B) purified rabbit IgG as an isotype control. The first column of panels displays DAPI staining for nuclei. The second column of panels displays M44 staining, marking MCMV replication compartments. The third column of panels displays staining H3K27me3 or isotype IgG. The fourth column of panels displays a merged image of the first three channels. The fifth column of panels displays a high-magnification image of the merged image from the fourth column. All images are 40×, unless otherwise indicated.
(TIF)
