# Supplementary figures and images for: Polycomb Repressive Complex 2 Targets Murine Cytomegalovirus Chromatin for Modification and Associates with Viral Replication Centers
Source: PLoS One. 2012 Jan 18;7(1):e29410. doi: 10.1371/journal.pone.0029410 (PMC3261147; doi:10.1371/journal.pone.0029410)

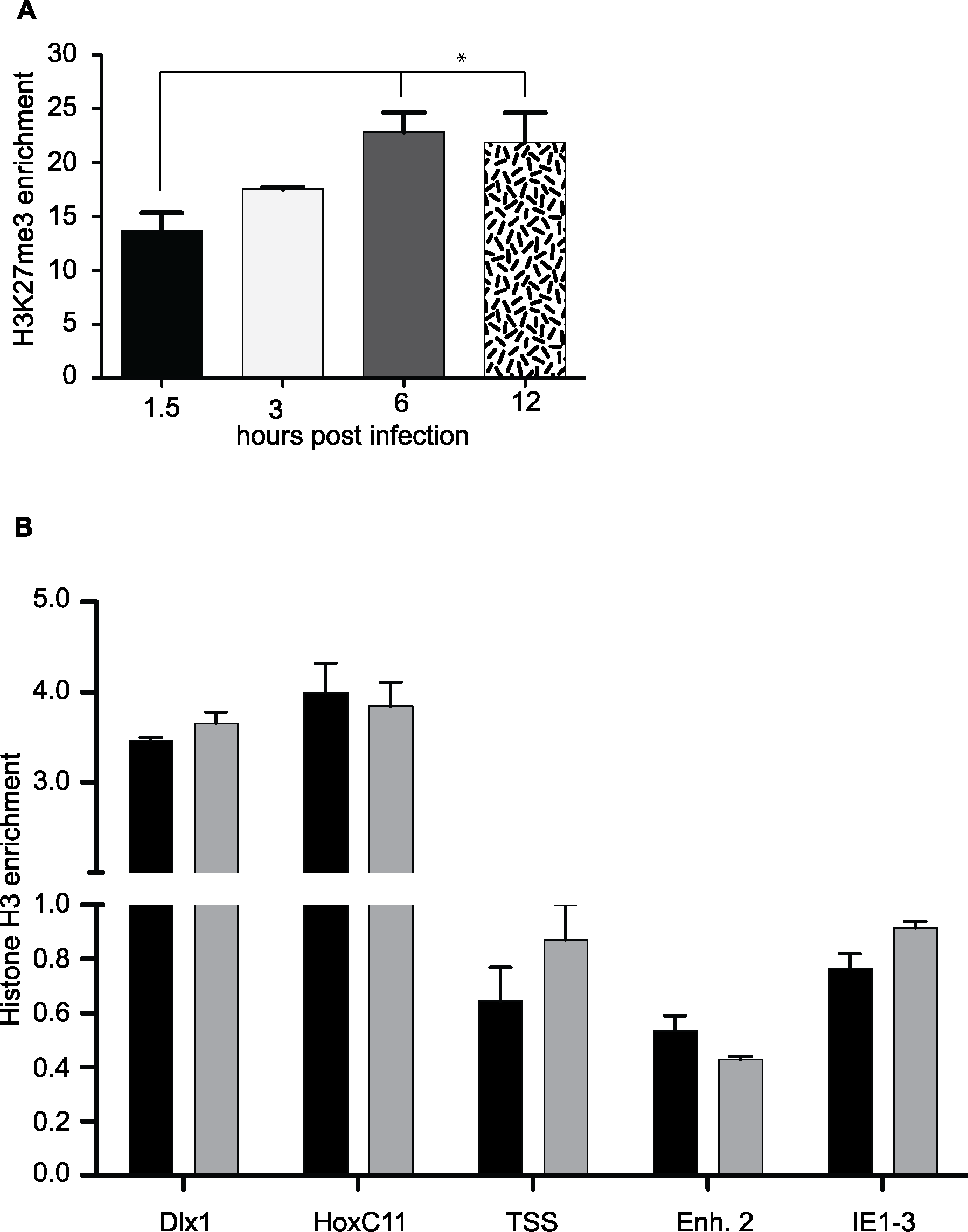

Supplement: Figure S1 — ChIP analysis of (A) H3K27me3 enrichment at the HoxC11 locus and (B) histone H3 at the MIE locus during MCMV infection. H3K27me3 enrichment was measured in mouse fibroblasts from 1.5 to 12 hpi. (* P value<0.05, ANOVA). ChIPs using anti-H3 antibody were analyzed by Q-PCR using primer/probe sets specific for the indicated loci at 1.5 (black bars) and 3 hpi (gray bars) as indicated. Each graph displays the mean value and S.E.M. for each queried locus, with data from two independent ChIPs. The results are presented as the IgG subtracted %Input of the queried locus normalized to the IgG subtracted β-Actin %Input. (TIF) [file pone.0029410.s001.tif]

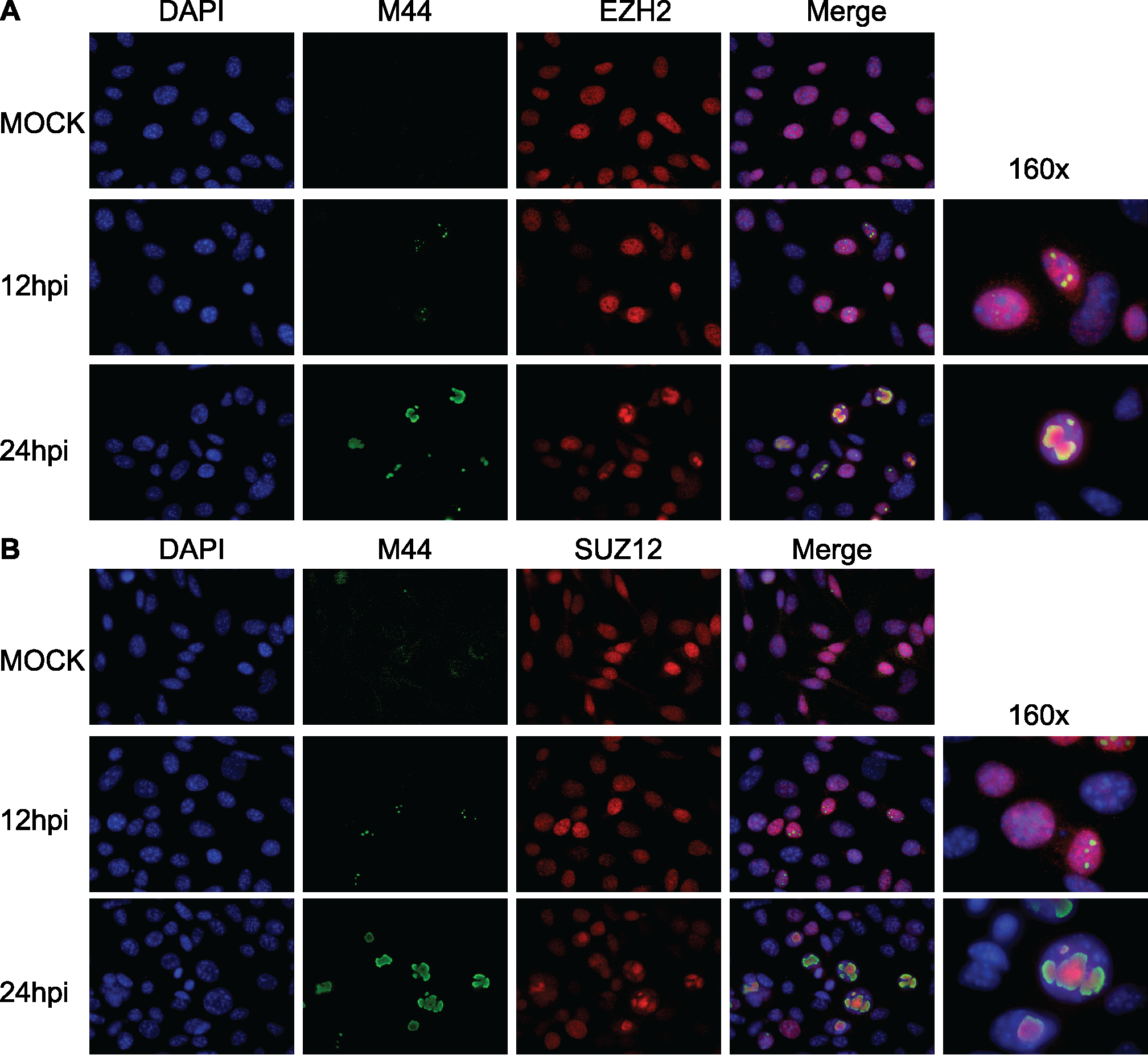

Supplement: Figure S2 — Co-immunofluorescence assay for PRC2 enrichment within MCMV replication compartments. At 12 and 24 hpi, mock-infected or MCMV-infected fibroblasts were fixed and incubated with antibodies against M44 and (A) EZH2 or (B) SUZ12. The first column of panels displays DAPI staining for nuclei. The second column of panels displays M44 staining, marking MCMV replication compartments. The third column of panels displays staining EZH2 or SUZ12. The fourth column of panels displays a merged image of the first three channels. The fifth column of panels displays a high-magnification image of the merged image from the fourth column. All images are 40×, unless otherwise indicated. (TIF) [file pone.0029410.s002.tif]

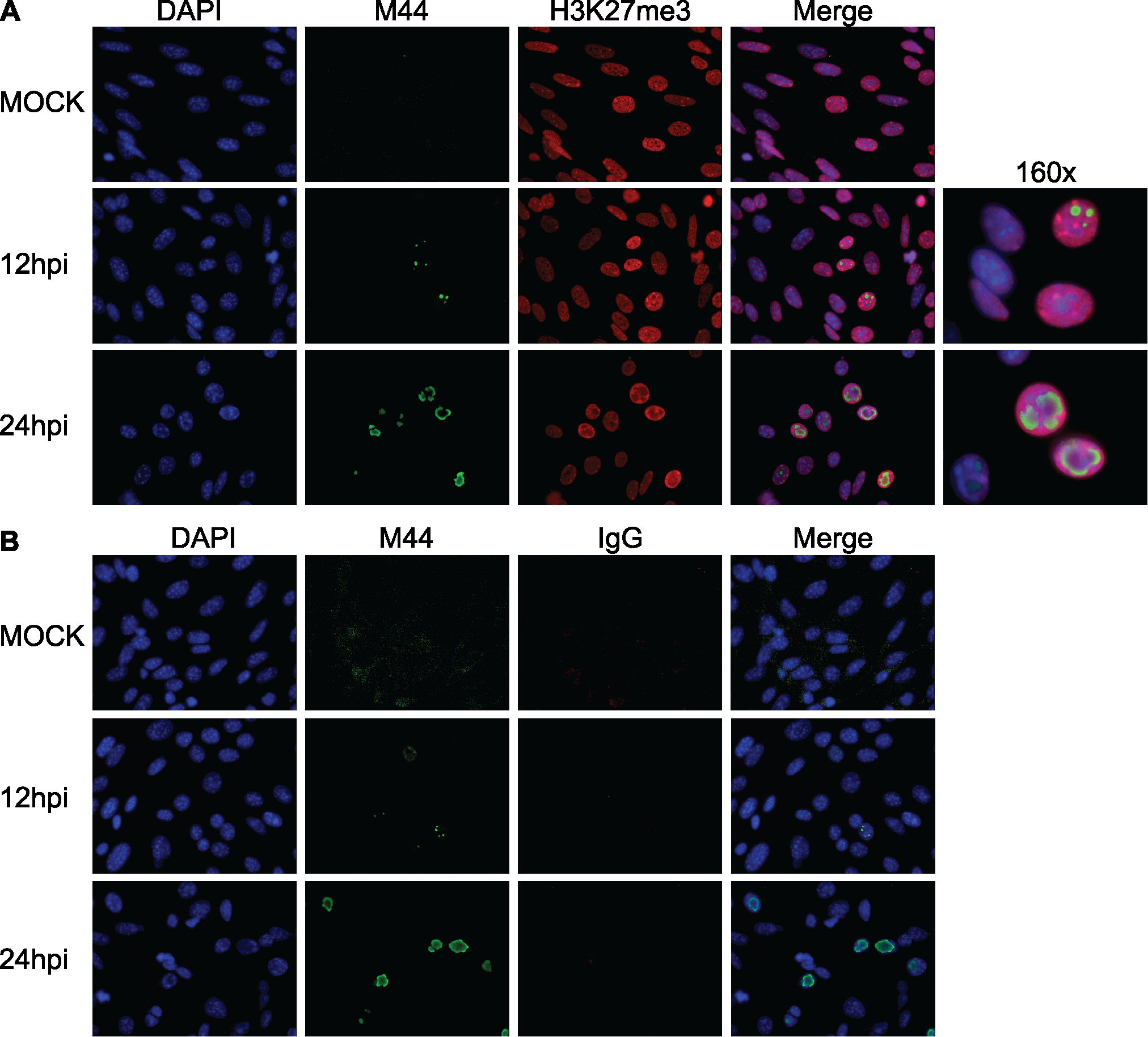

Supplement: Figure S3 — Co-immunofluorescence assay for PRC2 enrichment within MCMV replication compartments. At 12 and 24 hpi, mock-infected or MCMV-infected fibroblasts were fixed and incubated with antibodies against M44 and (A) H3K27me3 or (B) purified rabbit IgG as an isotype control. The first column of panels displays DAPI staining for nuclei. The second column of panels displays M44 staining, marking MCMV replication compartments. The third column of panels displays staining H3K27me3 or isotype IgG. The fourth column of panels displays a merged image of the first three channels. The fifth column of panels displays a high-magnification image of the merged image from the fourth column. All images are 40×, unless otherwise indicated. (TIF) [file pone.0029410.s003.tif]
